# Supplementary material for: Morphometric and taxonomic approach to describe Heterospio variabilis (Annelida, Longosomatidae), a new species with three size-dependent morphotypes, from the Gulf of California, Eastern Pacific
Source: PeerJ. 2024 Apr 4;12:e17093. doi: 10.7717/peerj.17093 (PMC10999154; doi:10.7717/peerj.17093)
Supplement: Supplemental Information 1 [file peerj-12-17093-s001.docx]

**Table S1:**

**Descriptive statistics to the 11 characters used in the morphometric analysis.**

| Variables | Valid N | Mean | Minimum | Maximum | Standard Deviation | Coeficient of Variation |
| --- | --- | --- | --- | --- | --- | --- |
| Total length | 56 | 10.91 | 5.20 | 19.87 | 3.53 | 32.35 |
| Number of branchiae | 56 | 6.57 | 4.00 | 8.00 | 1.22 | 18.55 |
| Prostomium length | 56 | 0.28 | 0.14 | 0.53 | 0.08 | 27.66 |
| Prostomium width | 56 | 0.32 | 0.16 | 0.50 | 0.08 | 24.30 |
| Length CH1-CH8 | 56 | 2.92 | 1.62 | 4.49 | 0.71 | 24.16 |
| Anterior width | 56 | 0.54 | 0.20 | 0.95 | 0.16 | 30.39 |
| Length CH9 | 56 | 0.41 | 0.13 | 0.98 | 0.17 | 40.95 |
| Length CH10 | 56 | 1.62 | 0.60 | 4.23 | 0.72 | 44.17 |
| Length CH11 | 56 | 2.72 | 0.80 | 7.75 | 1.40 | 51.60 |
| Length CH12 | 56 | 2.95 | 0.05 | 8.00 | 1.65 | 55.84 |
| Rate ch9L/Anterior region | 56 | 0.13 | 0.06 | 0.29 | 0.05 | 38.95 |
