# Supplementary material for: Morphometric and taxonomic approach to describe Heterospio variabilis (Annelida, Longosomatidae), a new species with three size-dependent morphotypes, from the Gulf of California, Eastern Pacific
Source: PeerJ. 2024 Apr 4;12:e17093. doi: 10.7717/peerj.17093 (PMC10999154; doi:10.7717/peerj.17093)
Supplement: Supplemental Information 2 — Significant values at p < .05 (N = 56) are showed in bold. Abbreviations are explained in the methodology section. [file peerj-12-17093-s002.docx]

**Table S2:**

**Pearson’s correlation among the 11 characters used in the morphometric analysis.** Significant values at *p* < .05 (N=56) are showed in bold. Abbreviations are explained in the methodology section.

| Variables | tL12 | brN | prL | prW | ch1-ch8L | anW | ch9L | ch10L | ch11L | ch12L | Rch9/anL |
| --- | --- | --- | --- | --- | --- | --- | --- | --- | --- | --- | --- |
| Total length | 1.00 |  |  |  |  |  |  |  |  |  |  |
| Number of branchiae | **0.53** | 1.00 |  |  |  |  |  |  |  |  |  |
| Prostomium length | 0.44 | **0.68** | 1.00 |  |  |  |  |  |  |  |  |
| Prostomium width | 0.41 | **0.57** | **0.67** | 1.00 |  |  |  |  |  |  |  |
| Length CH1-CH8 | 0.45 | **0.49** | 0.25 | 0.32 | 1.00 |  |  |  |  |  |  |
| Anterior width | **0.56** | **0.81** | **0.73** | **0.66** | 0.39 | 1.00 |  |  |  |  |  |
| Length CH9 | **0.48** | **0.68** | **0.77** | **0.55** | 0.41 | **0.78** | 1.00 |  |  |  |  |
| Length CH10 | **0.79** | 0.47 | **0.50** | 0.40 | 0.33 | **0.57** | **0.56** | 1.00 |  |  |  |
| Length CH11 | **0.86** | 0.37 | 0.33 | 0.33 | 0.16 | 0.42 | 0.41 | **0.78** | 1.00 |  |  |
| Length CH12 | **0.80** | 0.30 | 0.21 | 0.19 | 0.21 | 0.32 | 0.12 | 0.38 | **0.52** | 1.00 |  |
| Rate ch9L/Anterior region | 0.30 | **0.53** | **0.69** | 0.39 | -0.13 | **0.65** | **0.82** | 0.43 | 0.40 | 0.06 | 1.00 |
