# Supplementary material for: Morphometric and taxonomic approach to describe Heterospio variabilis (Annelida, Longosomatidae), a new species with three size-dependent morphotypes, from the Gulf of California, Eastern Pacific
Source: PeerJ. 2024 Apr 4;12:e17093. doi: 10.7717/peerj.17093 (PMC10999154; doi:10.7717/peerj.17093)
Supplement: Supplemental Information 3 [file peerj-12-17093-s003.docx]

**Table S3:**

**Eigenvalues and Eigenvectors of the PCA to the 11 characters used in the morphometric analysis.**

*Eigenvalues*

| Principal Component | Eigenvalues | %Variation | Cumulative %Variation |
| --- | --- | --- | --- |
| 1 | 5.92 | 53.8 | 53.8 |
| 2 | 1.81 | 16.5 | 70.3 |

*Eigenvectors* (Coeficients in the linear combinations of variables making up PC’s)

| Variable | PC1 | PC2 |
| --- | --- | --- |
| Total length | -0.33 | 0.43 |
| Number of branchiae | -0.33 | -0.14 |
| Prostomium length | -0.33 | -0.28 |
| Prostomium width | -0.29 | -0.17 |
| Length CH1-CH8 | -0.19 | 0.14 |
| Anterior width | -0.36 | -0.17 |
| Length CH9 | -0.35 | -0.28 |
| Length CH10 | -0.32 | 0.23 |
| Length CH11 | -0.28 | 0.37 |
| Length CH12 | -0.20 | 0.50 |
| Rate ch9L/Anterior region | -0.28 | -0.36 |
