# Supplementary material for: Morphometric and taxonomic approach to describe Heterospio variabilis (Annelida, Longosomatidae), a new species with three size-dependent morphotypes, from the Gulf of California, Eastern Pacific
Source: PeerJ. 2024 Apr 4;12:e17093. doi: 10.7717/peerj.17093 (PMC10999154; doi:10.7717/peerj.17093)
Supplement: Supplemental Information 4 [file peerj-12-17093-s004.docx]

**Table S4:**

**Selected variables by the Discriminant Analysis (F_(12, 96)_= 18.2; *p*< 0.0001) to separate the three morphotypes of *Heterospio variabilis* sp. nov.**

| Variable | Wilks' Partial Lambda | F-remove | *p*-level |
| --- | --- | --- | --- |
| Number of branchiae | 0.57 | 18.07 | 0.000 |
| Wide anterior region | 0.73 | 8.82 | 0.001 |
| Prostomium width | 0.87 | 3.48 | 0.039 |
| Rate ch9L/Anterior region | 0.84 | 4.42 | 0.017 |
| Length CH1-CH8 | 0.82 | 5.30 | 0.008 |
| Length CH9 | 0.86 | 3.99 | 0.025 |
