# Supplementary material for: Morphometric and taxonomic approach to describe Heterospio variabilis (Annelida, Longosomatidae), a new species with three size-dependent morphotypes, from the Gulf of California, Eastern Pacific
Source: PeerJ. 2024 Apr 4;12:e17093. doi: 10.7717/peerj.17093 (PMC10999154; doi:10.7717/peerj.17093)
Supplement: Supplemental Information 5 [file peerj-12-17093-s005.docx]

**Table S5:**

**Standardized coefficients for canonical variables.** The highest weights are showed in bold.

| Variable | Root 1 | Root 2 |
| --- | --- | --- |
| Number of branchiae | **0.751** | -0.123 |
| Wide anterior region | 0.369 | **1.237** |
| Prostomium width | 0.046 | -0.825 |
| Rate ch9L/Anterior region | **1.673** | **-2.509** |
| Length CH1-CH8 | **1.237** | **-1.568** |
| Length CH9 | **-1.563** | **1.674** |
| Eigenval | 6.812 | 0.373 |
| Cumulative proportion | 0.948 | 1.000 |
