# Supplementary material for: Morphometric and taxonomic approach to describe Heterospio variabilis (Annelida, Longosomatidae), a new species with three size-dependent morphotypes, from the Gulf of California, Eastern Pacific
Source: PeerJ. 2024 Apr 4;12:e17093. doi: 10.7717/peerj.17093 (PMC10999154; doi:10.7717/peerj.17093)
Supplement: Supplemental Information 6 [file peerj-12-17093-s006.docx]

**Table S6:**

**Eigenvalues and Eigenvectors of the PCA to the environmental condition on the three morphotypes found.**

*Eigenvalues*

| PC | Eigenvalues | % Variation | Cumulative % Variation |
| --- | --- | --- | --- |
| 1 | 3.67 | 61.8 | 61.8 |
| 2 | 1.2 | 20.2 | 82 |

*Eigenvectors* (Coefficients in the linear combinations of variables making up PC’s)

| Variable | PC1 | PC2 |
| --- | --- | --- |
| Depth (m) | 0.484 | -0.157 |
| Salinity (psu) | -0.333 | -0.02 |
| Temperature (°C) | -0.122 | 0.872 |
| Dissolved Oxygen (ml/L) | -0.49 | -0.07 |
| Organic matter (%) | 0.439 | 0.442 |
| Sand (%) | -0.455 | 0.115 |
